# A Cadaveric Study of the Rotator Cable: Interrogating the Suspension Bridge Model

**DOI:** 10.7759/cureus.70795

**Published:** 2024-10-03

**Authors:** Timothy R Kanne, John Lusk, Cassidy Clark, Cody Jones, Leanna Kanne, Daniel Cawley

**Affiliations:** 1 Medicine, Edward Via College of Osteopathic Medicine-Auburn, Auburn, USA; 2 Education, Truett McConnell University, Cleveland, USA; 3 Anatomy, Edward Via College of Osteopathic Medicine-Auburn, Auburn, USA

**Keywords:** cadaveric study, shoulder pathology, infraspinatus, rotator cuff, rotator cable

## Abstract

Purpose

The objective of this cadaveric study was to describe the anatomical relationships between the rotator cuff muscles and the rotator cable.

Methods

In 30 formaldehyde-fixed shoulders from 20 cadavers, the rotator cuff and rotator cable were dissected and the glenohumeral joint opened. The orientation and attachments of the rotator cable to the rotator cuff muscles were described, and the severity of any osteoarthritis, labral pathology, and rotator cuff pathology present was documented. The width and thickness of the infraspinatus attachments to the rotator cable were measured.

Results

The infraspinatus muscle was noted to be more loosely adherent to the rotator cable, while the supraspinatus and teres minor were tightly adherent to the cable. Specifically, the superior-most portion of the infraspinatus was found to be less tightly adherent than the inferior-most portion in 26 of the 30 shoulders studied. The thickness/width ratio of the inferior-most portion of infraspinatus was significantly different in shoulders with more-than-minimal osteoarthritis and labral pathology (p=0.048 and p=0.041, respectively).

Conclusion

While the supraspinatus and teres minor muscles were tightly adherent to the cable in all shoulders, the degree of attachment of the superior-most portion of the infraspinatus muscle was notably less in 26 of the 30 shoulders studied. This could mean that only the inferior portion of the infraspinatus participates in stress shielding through the cable or be a compensatory response to increased load on the tendon. This work is expected to provide insight into the function of the rotator cable and the different functions of the infraspinatus.

## Introduction

While the morphology of the rotator cuff is well described in the literature, less is known about the morphology of the rotator cable.  The rotator cable has been described as a semicircular, fibrous, band-like thickening of the glenohumeral joint capsule that runs between the tubercles of the humerus, lacing together the capsular attachment of the supraspinatus, infraspinatus, and teres minor muscles [1-3].  Because of its orientation and thickness, the cable is believed to function as a suspension bridge, helping in evenly distributing the forces generated by the supraspinatus and infraspinatus muscles along the superior and posterior rotator cuff [3,4].  More specifically, it is analogous to a cable connecting two pillars of a suspension bridge as it transfers the tension generated along the rotator cuff muscles to the rotator cable’s bony attachments [5]. Therefore, the rotator cable is believed to preserve rotator cuff tendon function in the event of a tear, limiting tear progression and protecting the rotator crescent, an avascular, injury-prone area formed by the tendinous insertion of the infraspinatus and supraspinatus [6].  However, a study by Yuri et al. found a variable attachment pattern between the rotator cuff muscles and the glenohumeral capsule [7]. This indicates a need to revisit the suspension bridge model.

Pathological conditions of the shoulder are known to alter the morphology of the shoulder. Previous studies have shown that the rotator cuff can atrophy in response to glenohumeral osteoarthritis and that the progression of osteoarthritis is higher in patients with rotator cuff retears [8,9].  Changes in shoulder mechanics secondary to rotator cuff tears have been shown to play a significant role in pathological changes of the superior labrum [10]. Furthermore, the rotator cuff atrophies in response to tendon tears [11].  More in-depth studies of the supraspinatus have shown that in addition to atrophy of both type 1 and 2 muscle fibers, there is also a shift toward type 2 fibers, leading to a loss of muscle endurance and increased fatigability in the presence of rotator cuff tears [12]. Increased rotator cuff fatigability alters shoulder kinematics, possibly leading to increased stress on neighboring structures [13]. Additionally, the rotator cable itself has been shown to increase in thickness in the presence of glenohumeral capsule tears [14]. Characterization of the morphological changes in the rotator cable in the presence of rotator cuff and glenohumeral pathology may lead to a better understanding of its role in shoulder function and improve upon the suspension bridge model.

This study aims to further our knowledge of the anatomical and structural characteristics of the rotator cable by documenting its attachments to the supraspinatus, infraspinatus, and teres minor on cadaveric specimens and to investigate any differences in morphology of the rotator cuff-cable attachment in the presence of shoulder pathology. 

This article was previously posted to the Research Square preprint server on March 20, 2024.

## Materials and methods

The authors extend their heartfelt gratitude to the individuals who generously donated their bodies to science, enabling this anatomical research. The findings from this study have the potential to enhance our collective understanding and ultimately improve patient care.

Following a gross anatomy course, institutional approval was granted to perform anatomical studies on the cadaveric shoulders (the cadavers were donated with prior informed consent for research and education purposes, and institutional IRB policies and guidelines governing the ethical conduct of research involving human cadavers were observed). All formaldehyde-fixed cadaveric shoulders were initially included in the study. Shoulders were excluded from the study if dissections were unable to be completed due to desiccation or if prior dissection of the rotator cuff or glenohumeral joint had been performed. A total of 30 formaldehyde-fixed shoulders from 20 cadavers (11 female, 9 male, average age of 79.65 years) were dissected. 

Dissections were performed by removing the deltoid from its distal and proximal attachments.  The trapezius was detached from the clavicle and scapula and was reflected medially.  After removing all neurovascular structures connecting the shoulder to the neck and thorax, an oscillating autopsy saw was used to make a vertical cut through the medial clavicle just lateral to the sternoclavicular joint and a horizontal cut at the midshaft of the humerus such that the glenohumeral joint and scapula could be completely separated from the rest of the cadaver.  An additional cut was made at the lateral aspect of the scapular spine, allowing the acromion to be removed so that there was an unobstructed view of the posterior glenohumeral joint.  The supraspinatus, infraspinatus, and teres minor were identified, dissected, and reflected from medial to lateral such that their attachments to the glenohumeral capsule could be seen.  If bursa was present, it was reflected in the rotator cuff muscles. An attempt was made to identify the rotator cable to compare its relationship to the capsular attachment of the rotator cuff muscles.

The attachments of the supraspinatus, infraspinatus, and teres minor muscles in relation to the cable were noted, the width and thickness of the superior- and inferior-most portions of the infraspinatus attachments to the rotator cable were measured with a digital caliper (Mitutoyo CD-600 CSX, Aurora, IL, USA) three times by the same person blinded to previous measurements, and the mean was calculated.  

A vertical incision superficial to the posterior glenohumeral joint was then made in the glenohumeral joint capsule from proximal to distal, and the joint capsule was opened. After opening the glenohumeral joint capsule posteriorly, the severity of any grossly visible osteoarthritis, labral pathology, and rotator cuff pathology was documented.

In the absence of a standardized way to classify grossly visible cadaveric joint pathology, we developed a rubric for classifying labral pathology, osteoarthritis, and rotator cuff pathology. Labral and osteoarthritis pathology classification was based on the approximate percentage of the total surface area of the glenohumeral joint or glenoid labrum that demonstrated anything that appeared abnormal to visual inspection, including, but not limited to, fraying, osteophytes, erosion, and calcifications. Glenohumeral joints with any bony or labral abnormalities present covering approximately less than one-third of the total glenohumeral joint or labrum surface area were classified as none/minimal. Joints with bony or labral abnormalities covering approximately more than one-third of the total surface area were classified as more-than-minimal (MTM). If present, rotator cuff pathology, including, but not limited to, fraying, tearing, and calcifications, was classified as none/minimal if affecting approximately less than one-third of the total thickness of the tendon or muscle and as MTM if affecting approximately more than one-third of the tendon or muscle thickness. To account for the size difference between cadavers, the thickness/width ratio was calculated for the superior and inferior portion of the infraspinatus’ attachment to the rotator cable for each shoulder. All classifications of pathology were documented and correlated with infraspinatus dimensions.

Analyses began by calculating sample mean and sample standard deviation thickness/width ratio to measure central location and dispersion of the ratio variable. These calculations were conducted by group. Due to the small sample size and lack of ability to adequately judge the assumptions of normality, all tests were conducted using the Exact Wilcoxon rank sum test. All analyses were conducted using SAS 9.4 (SAS Institute Inc., Cary, NC).

## Results

**Figure 1:**
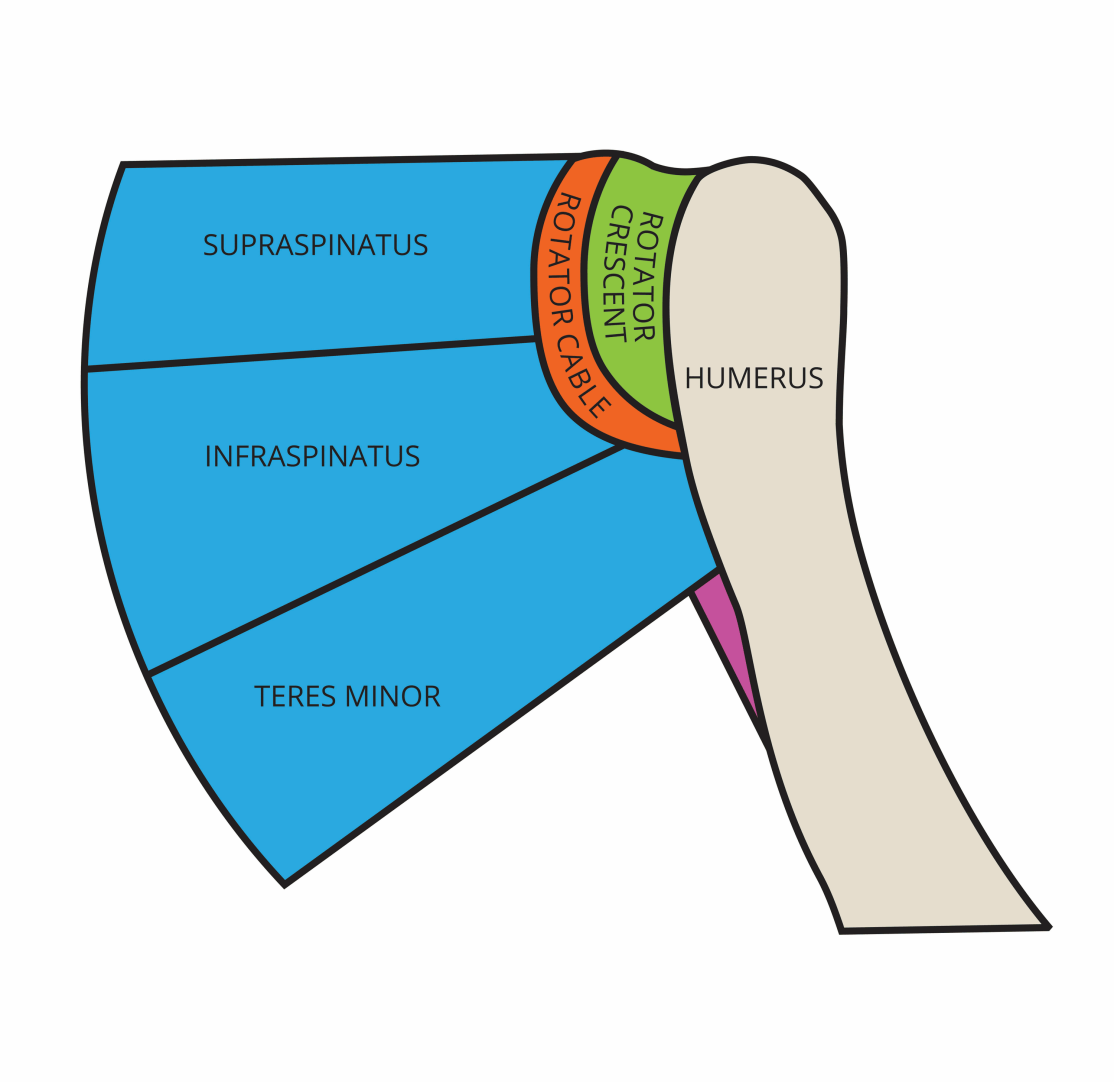
Posterior view of the right rotator cuff showing the rotator cable’s location. As described by Park and Jung and Burkhart et al. [5,6]. Figure drawn by author based on Asghar et al. [1].

**Figure 2:**
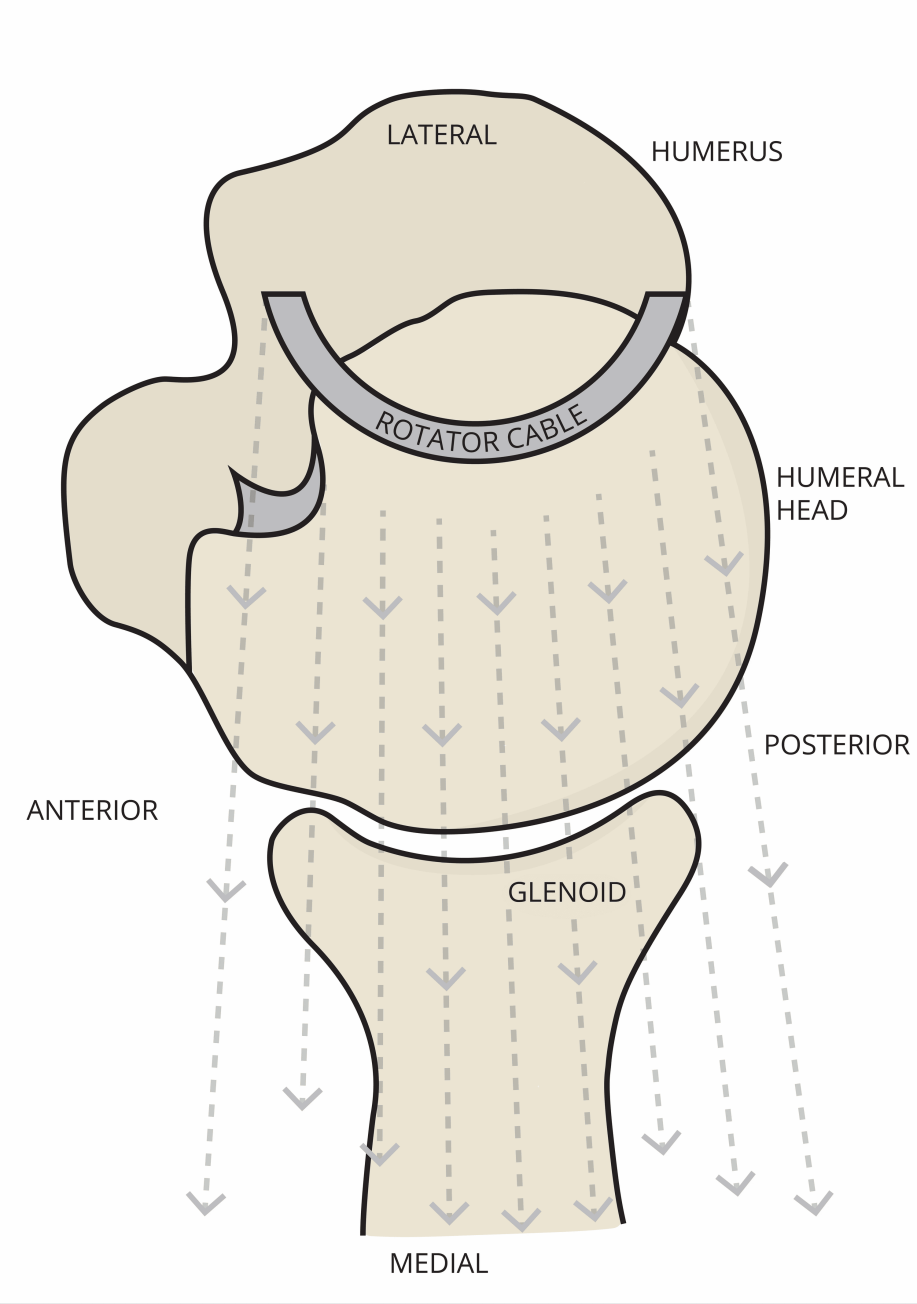
Superior view of the glenohumeral joint depicting how the rotator cable transmits abduction force. As described by Park and Jung and Burkhart et al. [5,6].  Figure drawn by the author based on Park and Jung [5].

**Figure 3:**
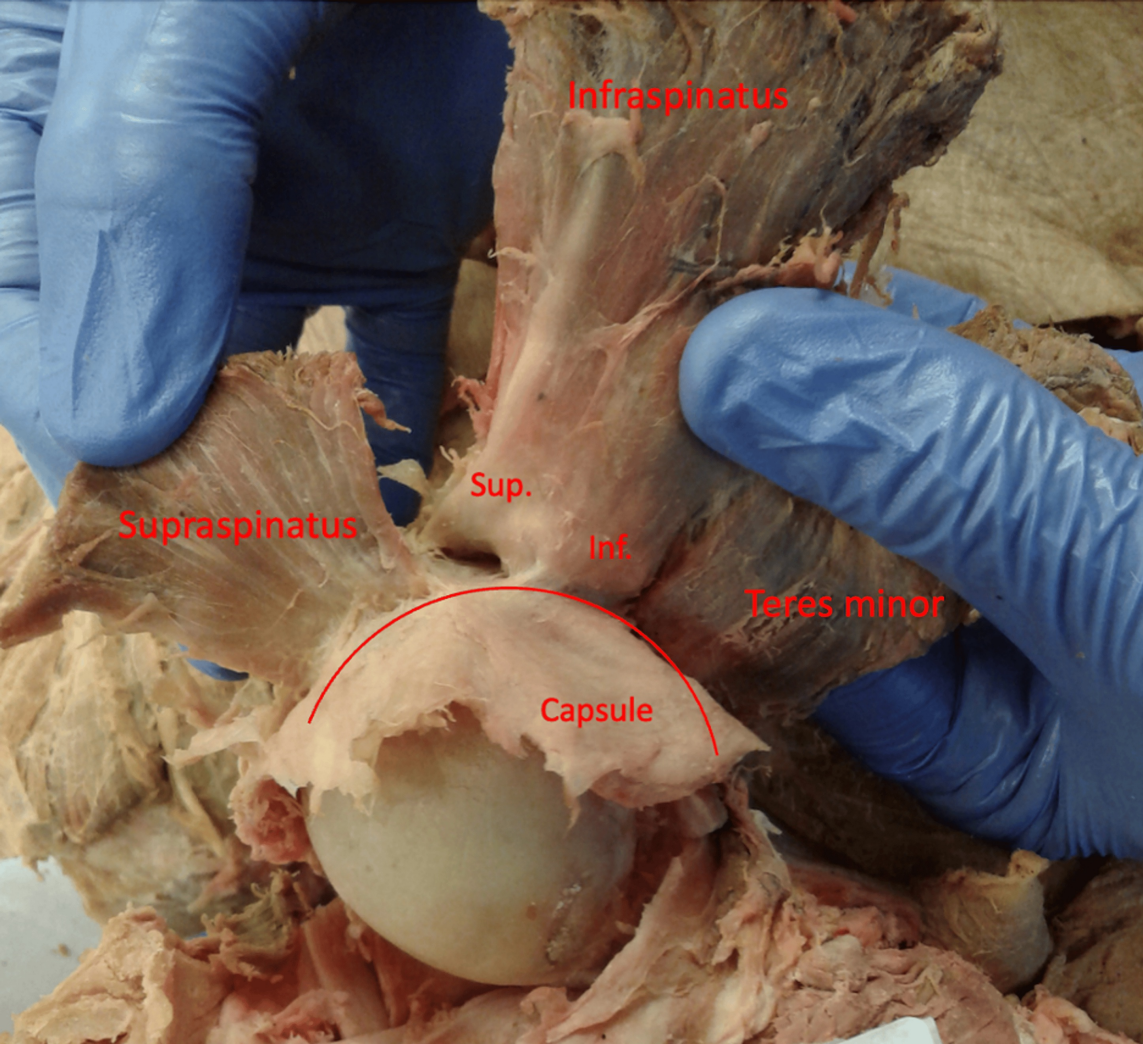
Posterior view of a right shoulder demonstrating the rotator cuff's attachments to the rotator cable. The red line overlay indicates the approximate superficial surface of the rotator cable. Sup., superior portion of infraspinatus; Inf., inferior portion of infraspinatus

**Figure 4:**
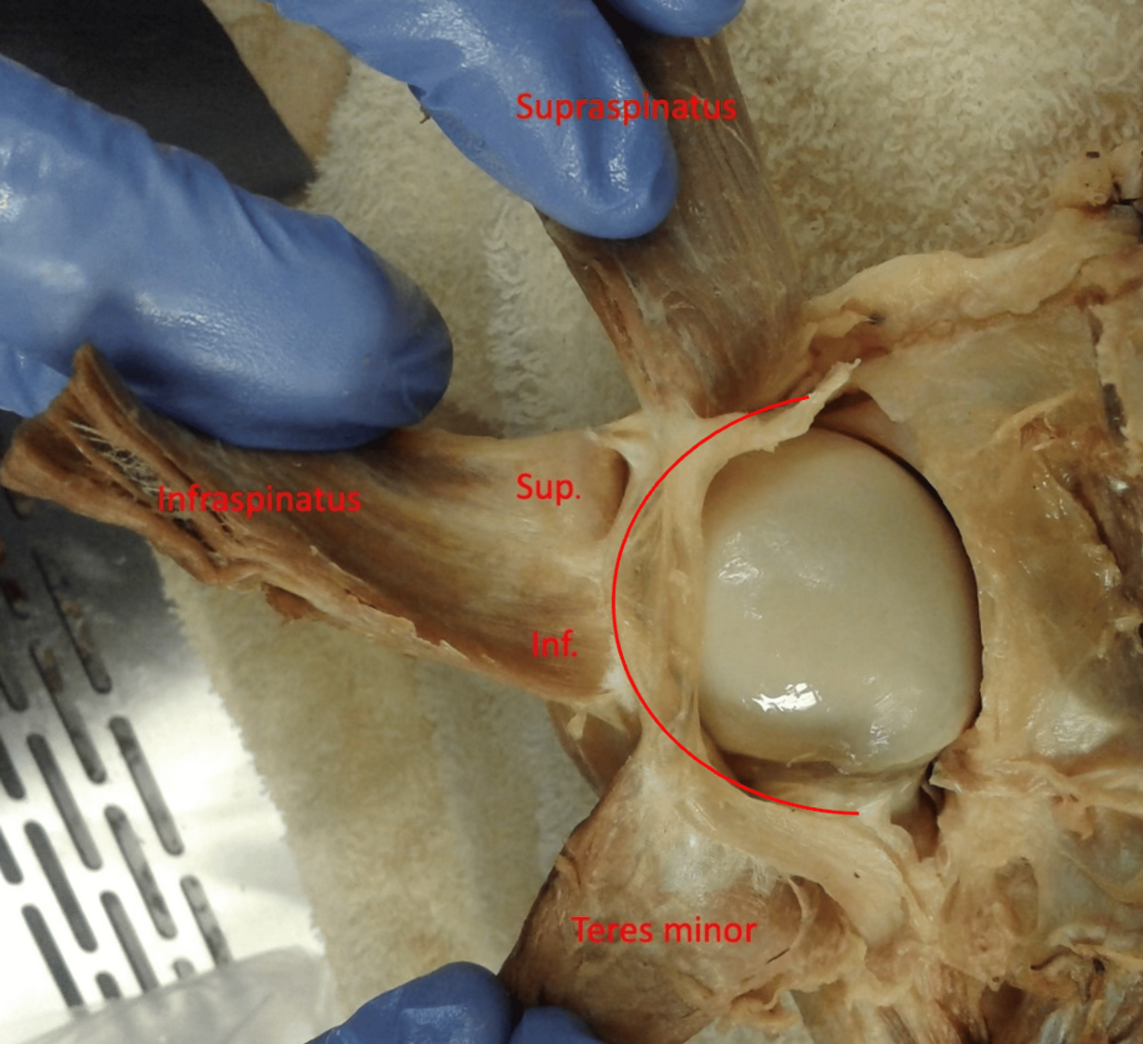
Posterior view of a left shoulder demonstrating the rotator cuff's attachments to the rotator cable. The red line overlay indicates the approximate superficial surface of the rotator cable. Sup., superior portion of infraspinatus; Inf., inferior portion of infraspinatus

During dissection of the rotator cable (Figures 1, 2), it was apparent that the infraspinatus had varying degrees of attachment to the rotator cable.  The superior-most portion of the infraspinatus had a different degree of attachment than the inferior-most portion of the infraspinatus when viewing the cable with the supraspinatus, infraspinatus, and teres minor muscles reflected laterally.  The superior-most portion of the infraspinatus was significantly less adherent to the rotator cable and the inferior-most portion of the infraspinatus was significantly more adherent to the rotator cable in 26 of the 30 (86%) shoulders dissected (Figures 3, 4).

Out of the 30 shoulders, MTM labral pathology was noted in 11, MTM osteoarthritis in 10, and MTM rotator cuff pathology in 4 (Table 1). A statistically significant difference was noted in the inferior infraspinatus thickness/width ratio in the MTM osteoarthritis and labral pathology groups compared to the none/minimal group (Table 2).

**Table 1:** Number of shoulders in each category of pathology.

Type of pathology	More than minimal	None/minimal
Cuff pathology	4	26
Osteoarthritis	10	20
Labral pathology	11	19

**Table 2:** Ratio variable of the superior and inferior portions of the infraspinatus compared between the two groups of pathology. All tests were conducted using the exact Wilcoxon rank sum test.

Position	More than minimal		None/minimal		P-value
-	N	Mean ± SD	N	Mean ± SD	-
Cuff pathology: superior infraspinatus	4	0.32 ± 0.13	26	0.21 ± 0.05	0.0769
Cuff pathology: inferior infraspinatus	4	0.30 ± 0.13	26	0.17 ± 0.05	0.1795
Osteoarthritis: superior infraspinatus	10	0.24 ± 0.05	20	0.23 ± 0.09	0.2714
Osteoarthritis: inferior infraspinatus	10	0.24 ± 0.12	20	0.17 ± 0.04	0.0477
Labral pathology: superior infraspinatus	11	0.24 ± 0.05	19	0.23 ± 0.09	0.1066
Labral pathology: inferior infraspinatus	11	0.24 ± 0.11	19	0.17 ± 0.05	0.0409

## Discussion

During our dissections, we noted an interesting relationship between shoulder pathology and a pattern in the infraspinatus-rotator cable attachment that has not been previously described. Specifically, the attachment pattern found could signify that only the inferior portion of the infraspinatus participates in stress shielding through the cable, or it could be a compensatory response to increased load on the tendon, such as that from glenohumeral pathology [5,15]. Mura et al.'s findings, which showed that the infraspinatus could generate abduction torque even when the entire superior infraspinatus was torn, suggest that the cable could play a role in keeping the infraspinatus engaged with the rest of the cuff during abduction, even in the setting of large tears [15]. Alternatively, the difference in attachment could simply be the effect of the different partitions of the infraspinatus muscle belly [3]. In fact, a recent study by Yuri et al. found variation in how the infraspinatus attached to the rotator cable, specifically in the middle partition, which was firmly attached with dense connective tissue, while the rest of the infraspinatus had loose, less dense connective tissue [7]. While we did not note three separate partitions of the infraspinatus at the rotator cable, the variation we found may be attributed to different partitions of the infraspinatus and described differently by Yuri et al. Regardless of the nomenclature, our preliminary data suggest a statistically significant increase in the thickness/width ratio of the inferior portion of the infraspinatus in the presence of MTM osteoarthritis or labral pathology, which could be a sign of an increased stress load being transferred through the muscle, tendon, and rotator cable at its inferior attachment, leading to hypertrophy. This pattern of increased thickness/width ratio in the presence of significant pathology is consistent with what has been observed previously in the presence of glenohumeral capsule tears [14]. Our observations indicate the potential for the infraspinatus-rotator cable attachment to respond to pathological changes, indicating the need for further research to elucidate the exact role the rotator cable plays within the mechanics of the shoulder.

Similar observations to our infraspinatus-cable attachment findings have already been made about the cable’s superior-anterior attachment at the supraspinatus.  Detailed dissections have shown that the coracohumeral and anterosuperior glenohumeral ligaments merge with the rotator cable, providing the cable with additional anchoring and support superiorly and anteriorly [16-18].  Therefore, large anterior cable tears have been shown to cause a disproportionate amount of strain across the supraspinatus when compared to tears localized to the crescent area [19].  While the supraspinatus and anterior cable have received more attention in previous studies, we think our findings suggest that a similar pattern would be seen if the inferior portions of the cable were disrupted, which would presumably cause an increased amount of strain on the infraspinatus and teres minor muscles, and that repairing the inferior cable might improve surgical outcomes [6,10,20-22].  Our observed attachment pattern, which is consistent with previous supraspinatus-cable patterns observed, potentially confirms the cable's coupling role in the inferior part of the rotator cuff, where it has stronger attachments to the infraspinatus. 

This study is limited in several ways. Besides being a descriptive study, the attachment pattern observed in the infraspinatus could have been artificially augmented by the manner of dissection since the reflection of the rotator cuff muscles from medial to lateral could have separated some of the tissue connecting the infraspinatus to the rotator cable. Furthermore, there is no standardized way to classify gross cadaveric glenohumeral or rotator cuff pathology, and, thus, our pathology classification is somewhat arbitrary. With a different cut-off for disease severity, we may have found different relationships between rotator cable-infraspinatus attachment dimensions and disease severity. The cadavers’ medical history regarding the shoulder was unknown, and while no signs of prior shoulder surgery or injury on any of the dissected shoulders were noted, such signs could have been missed by visual inspection. Because it is unknown if there was a history of bursitis or other kinds of shoulder pathology, we are unable to determine if they affect how adherent the rotator cuff is to the rotator cable. Given the advanced age of the cadavers and their unknown prior physical activity history, the implications of our results could be limited to an older patient population and confounded by an uneven distribution of rotator cuff health. Finally, without knowing previous medical history and physical activity ability, we are unable to examine the relationship between the rotator cable's presence and rotator cuff functionality.

## Conclusions

In conclusion, our study of the morphology of the rotator cable-rotator cuff attachments suggests the morphology of the rotator cable changes in the presence of shoulder pathology. Future studies on the rotator cable should include histologic analysis of the rotator cable, particularly the inferior portion, and should aim to assess the relationship between the rotator cable’s presence and rotator cuff functionality. While there are surgical techniques that focus on repairing the anterior-superior rotator cable, our study suggests that repairing tears of the inferior rotator cable might improve surgical outcomes and might warrant consideration. While our study describes the morphology of the rotator cable-rotator cuff attachments and proposes potential surgical considerations for the inferior rotator cable, further investigations are necessary to enhance our understanding of the therapeutic potential of these findings in a more diverse patient population.
